# Supplementary material for: Switching the activity of Cas12a using guide RNA strand displacement circuits
Source: Nat Commun. 2019 May 7;10:2092. doi: 10.1038/s41467-019-09953-w (PMC6504869; doi:10.1038/s41467-019-09953-w)
Supplement: Supplementary file 1 — Supplementary Information [file 41467_2019_9953_MOESM1_ESM.pdf]

# Supplementary Information

## Switching the activity of Cas12a using guide RNA strand displacement circuits

Oesinghaus et al.

## Supplementary Figures

**Supplementary Figure 1 – Choice of targets for gRNAs**

**Supplementary Figure 2 – Additional target cutting assay for target sequence t2**

**Supplementary Figure 3 – Activation of handle-based SD gRNAs for a one-to-one ratio of target DNA and SD gRNA**

**Supplementary Figure 4 – Influence of 5' and 3' hairpins on the transcription of trigger RNAs**

**Supplementary Figure 5 – Data for different target-based SD gRNA designs**

**Supplementary Figure 6 – Supplementary fluorescence curves and bar graphs for target-based SD gRNAs and AND gates**

**Supplementary Figure 7 – Using SD gRNA AND gates to sense natural RNA sequences**

**Supplementary Figure 8 – Supplementary data for in vivo SD gRNA measurements**

**Supplementary Figure 9 – Analysis of crosstalk for in vivo SD gRNAs**

**Supplementary Figure 10 – Predicted folding (minimum free energy structures) of stem extension and 3' extension Cas12a gRNAs**

**Supplementary Figure 11 – Predicted folding of 5' extension Cas12a gRNAs**

**Supplementary Figure 12 – Predicted folding of selected in vitro SD gRNAs and triggers**

**Supplementary Figure 13 – Expected impact of GU base pairs on the design of SD gRNAs**

**Supplementary Figure 14 – Predicted folding of selected in vivo SD gRNAs and triggers**

## Supplementary Notes

**Supplementary Note 1 – Design of stem extension and 3' extension Cas12a gRNAs**

**Supplementary Note 2 – Design of 5' extension Cas12a gRNAs**

**Supplementary Note 3 – Design of in vitro SD gRNAs**

**Supplementary Note 4 – Transcription of RNA triggers**

**Supplementary Note 5 – Details of in vivo measurements**

**Supplementary Note 6 – Design of in vivo SD gRNAs**

## Supplementary Tables

**Supplementary Table 1 – Plasmid sequencing primers**

## Supplementary References

## Supplementary Figures

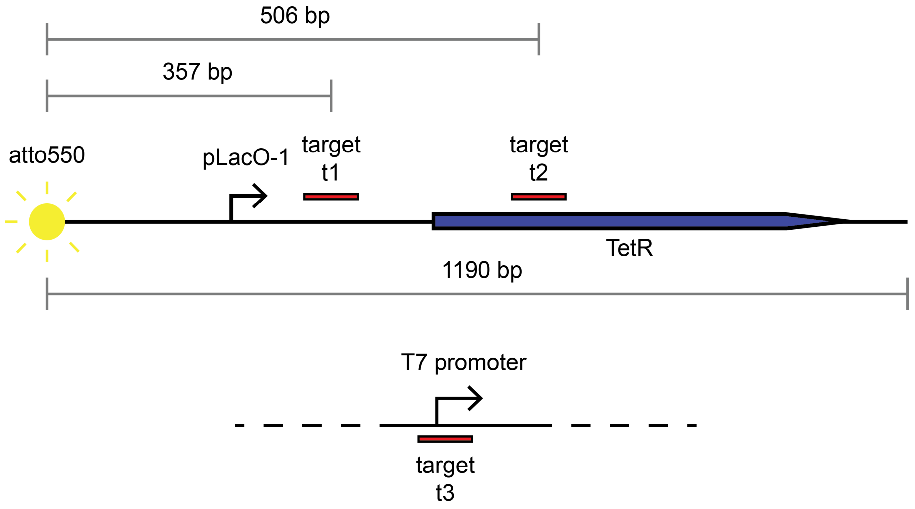


**Supplementary Figure 1. Choice of targets for gRNAs.** The most common target used for in vitro Cas12a cutting assays in this study has a length of 1190 nt and is PCR amplified from a plasmid containing the sequence for TetR with an atto550-labeled primer at the 5' end for imaging. Target t3 contains part of the sequence of the T7 promoter.

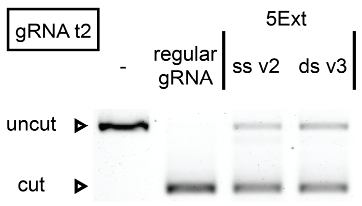


**Supplementary Figure 2. Additional target cutting assay for target sequence t2.** Agarose gel assaying target cutting by gRNAs extended at the 5' end for target sequence t2. (uncut: 1190 bp, cut: 506 bp) (5Ext: 5' extension, ss v2: single-stranded extension version 2, ds v3: double-stranded extension version 3)

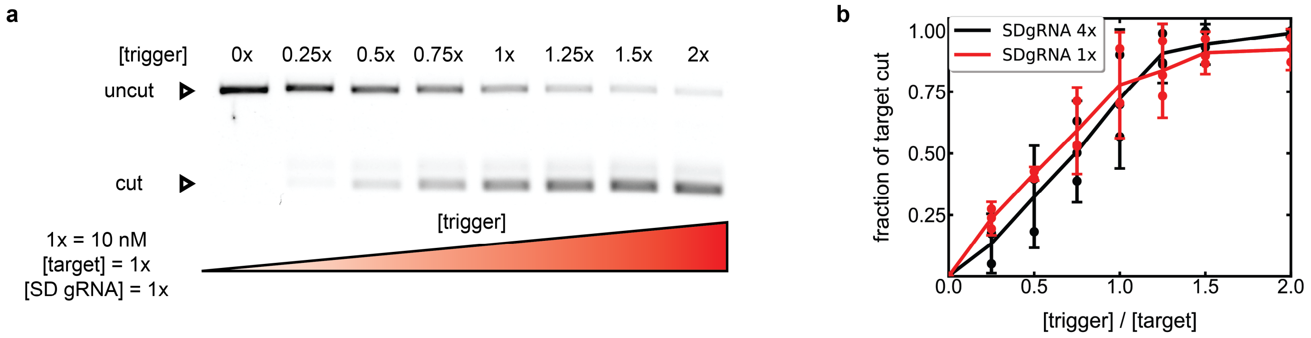


**Supplementary Figure 3. Activation of handle-based SD gRNAs for a one-to-one ratio of target DNA and SD gRNA.** (**a**) Agarose gel showing activation of target cutting by varying amounts of trigger RNA. (uncut: 1190 bp, cut: 357 bp) (**b**) Transfer function derived from the fraction of cut target by gels as shown in (a) for 1x and 4x SD gRNA concentration. There is only a small difference in activation for low trigger concentrations, but the cutting saturates below 100% cutting for 1x SD gRNA concentration. (N=3, t-distribution two-sided 90% confidence interval) Source data are provided as a Source Data file.

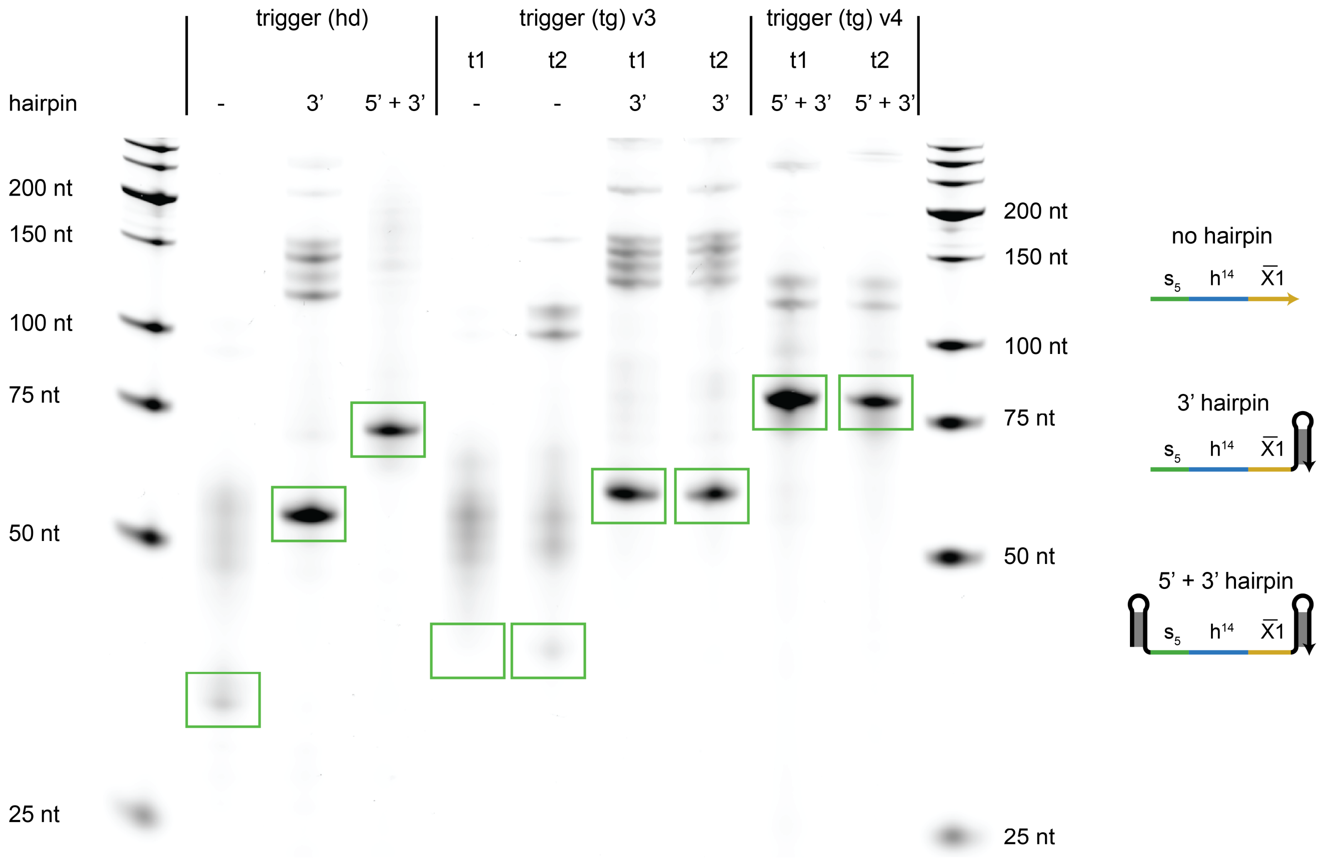


**Supplementary Figure 4. Influence of 5’ and 3’ hairpins on the transcription of trigger RNAs.** Denaturing PAGE showing the transcription of different trigger RNAs. The desired product is marked with a green box. The ladder is a Low Molecular Weight DNA ladder (NEB).

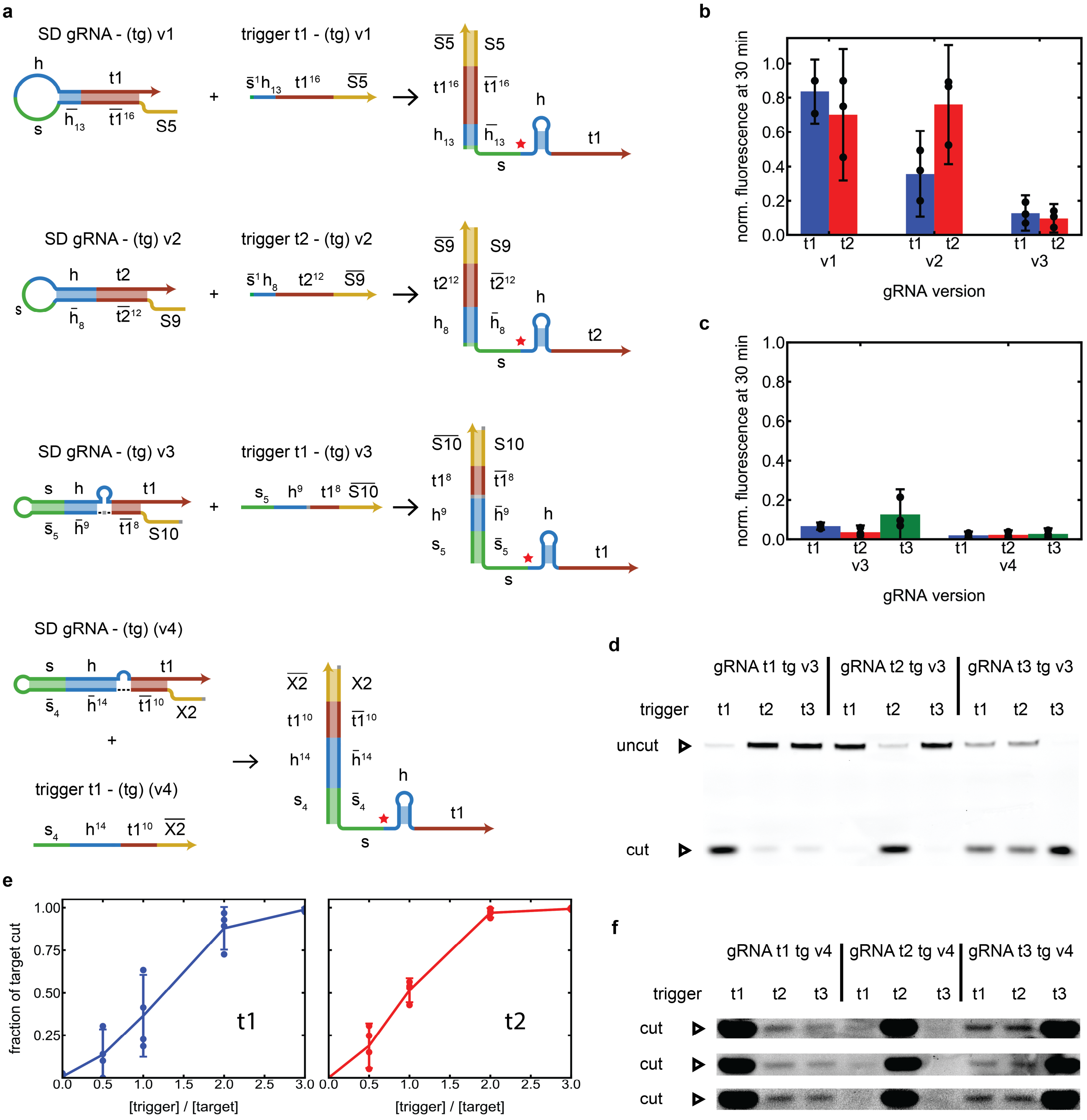


**Supplementary Figure 5. Data for different target-based SD gRNA designs.** (**a**) Designs of four different versions of target-based SD gRNAs. The last version (v4) is the one shown in the main text. (**b**) Fluorescence measurement using the ssDNase assay showing the leak of the first three version of target-based SD gRNAs in the absence of trigger for target sequences t1 and t2. (N=3, t-distribution two-sided 90% confidence interval) (**c**) Fluorescence measurement using the ssDNase assay showing the leak of versions v3 and v4 of target-based SD gRNAs in the absence of trigger for target sequences t1, t2 and t3. While the leak for target sequences t1 and t2 is comparatively low, there is significant leak for target sequence t3. The leak of version v4 SD gRNAs is minimal for all target sequences. (N=3, t-distribution two-sided 90% confidence interval) (**d**) Denaturing PAGE assaying target cutting by v3 of target-based SD gRNAs in the presence of different cognate and non-cognate triggers. While the target is cut in the presence of the cognate trigger, the intrinsic leak of this SD gRNA design leads to significant leaky cutting for target t3 and slightly leaky cutting for target t1. (uncut: 79 nt, cut: 22 nt) (**e**) Transfer function derived from the fraction of uncut target for different trigger concentrations for SD gRNAs t1 and t2 version 4. (N=4, t-distribution two-sided 90% confidence interval) (**f**) Individual gels comparing activation of target-based SD gRNAs (v4) for cognate and non-cognate triggers. Source data are provided as a Source Data file.

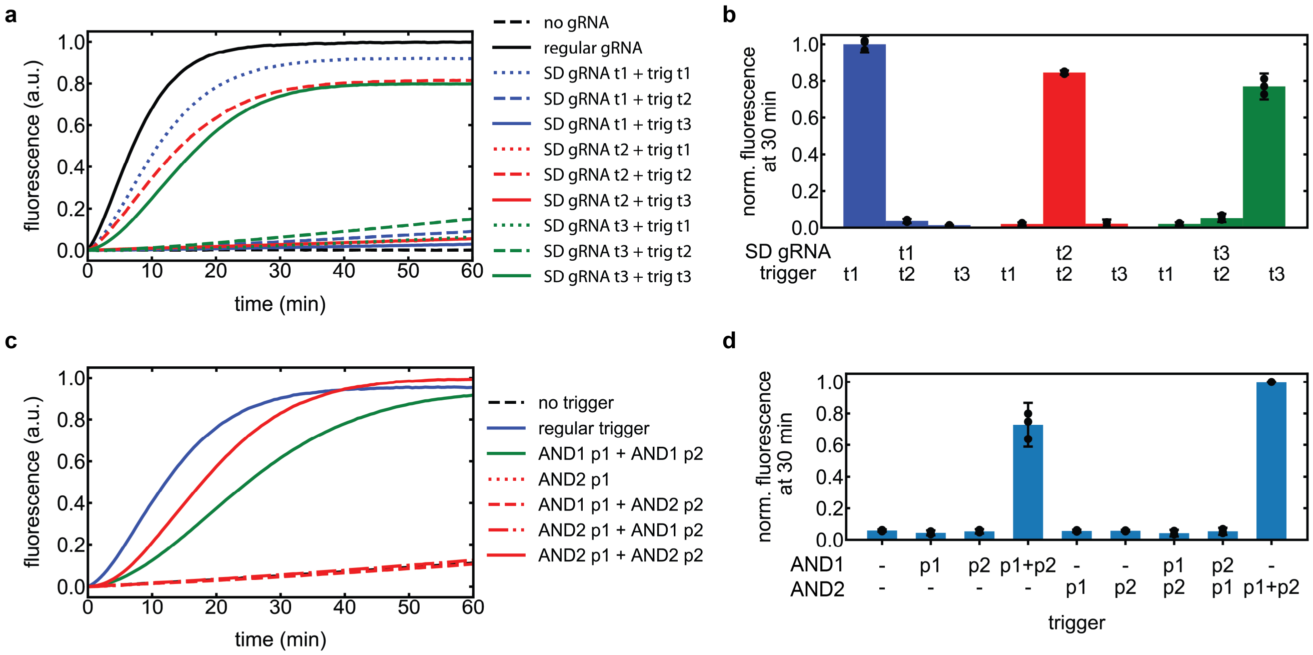


**Supplementary Figure 6. Supplementary fluorescence curves and bar graphs for target-based SD gRNAs and AND gates.** (**a**) Time trace of a fluorescence measurement of Cas12a ssDNase activity for target-based SD gRNAs in combination with different cognate and non-cognate triggers. (**b**) Fluorescence measured in (a) after 30 minutes of reaction time. (N=3, t-distribution two-sided 90% confidence interval) (**c**) Time trace of a fluorescence measurement of Cas12a ssDNase activity for a SD gRNA AND gate showing orthogonality based on the AND domain. (**d**) Fluorescence shown in (c) after 30 minutes of reaction time (N=3, t-distribution two-sided 90% confidence interval). Source data are provided as a Source Data file.

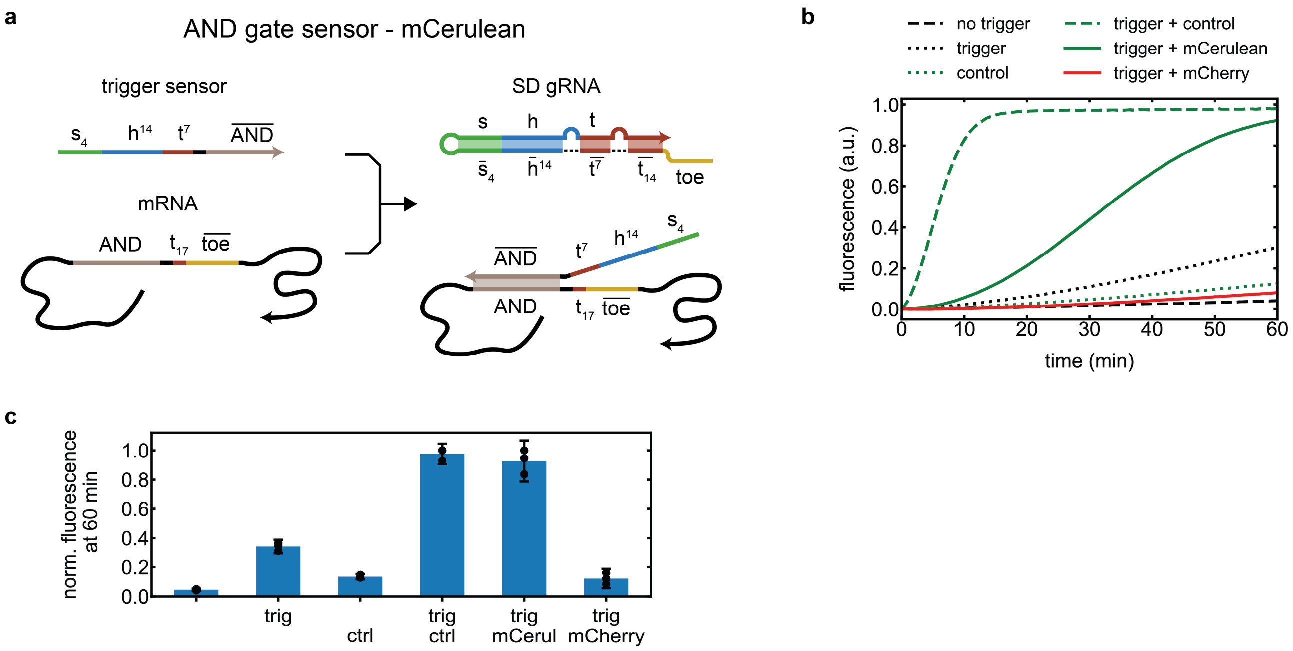


**Supplementary Figure 7. Using SD gRNA AND gates to sense natural RNA sequences.** (**a**) Design of an SD gRNA AND gate sensor for mRNAs. (**b**) Time trace of a fluorescence measurement of Cas12a ssDNase activity for an SD gRNA AND gate sensor for mCerulean mRNA. The control is an RNA containing only the sensed subsequence of the mCerulean mRNA. mCherry mRNA is used as a negative control. The measurement uses 60 nM Cas12a, 10 nM SD gRNA, and 37.5 nM trigger and mRNA input. (**c**) Fluorescence measured in (a) after 60 minutes of reaction time. trig = trigger, ctrl = control, mCerul = mCerulean mRNA, mCherry = mCherry mRNA (N=3, t-distribution two-sided 90% confidence interval). Source data are provided as a Source Data file.

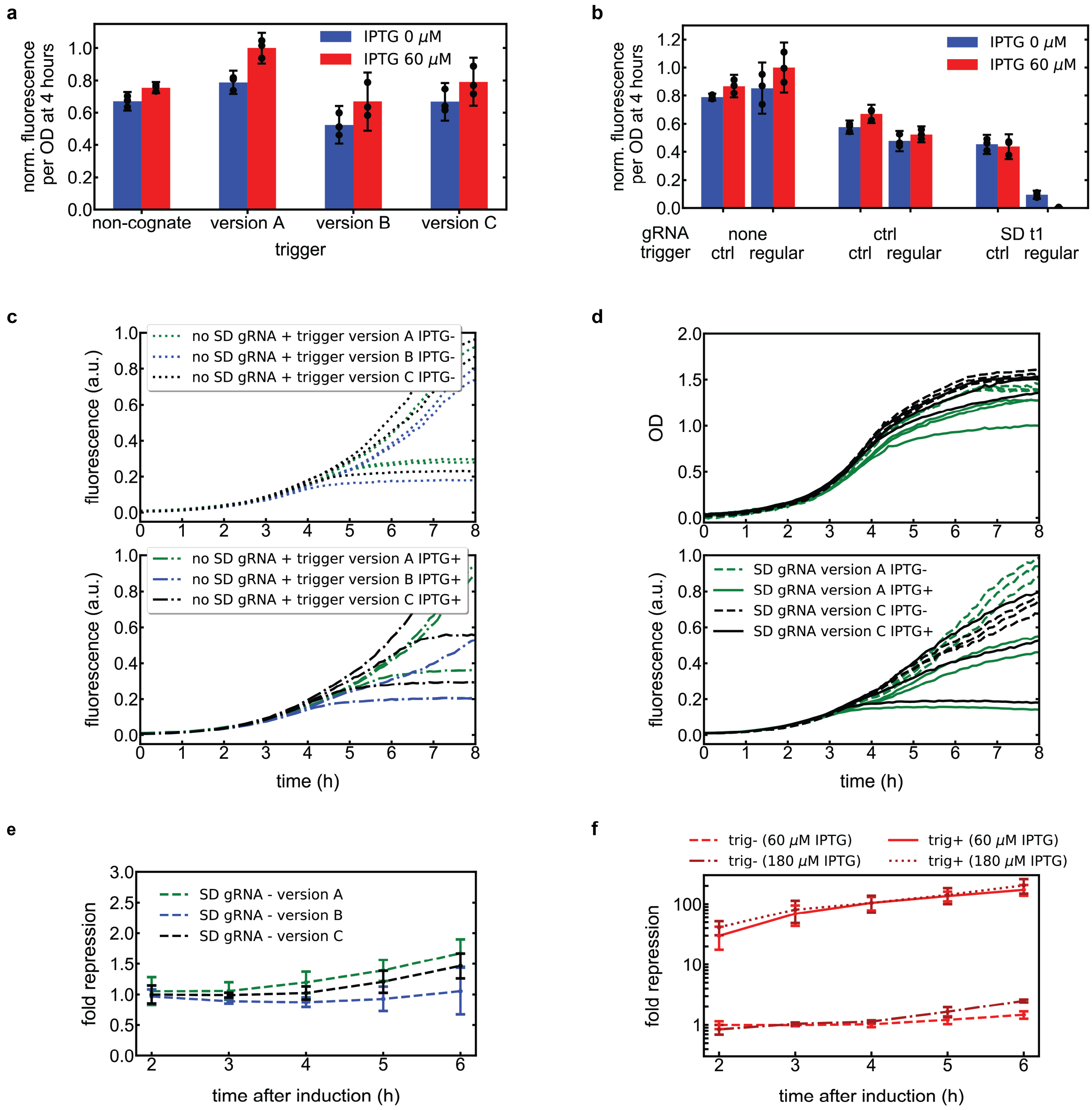


**Supplementary Figure 8. Supplementary data for in vivo SD gRNA measurements.** (**a**) Fluorescence at 4 hours for a pSB3C5 plasmid containing no gRNA with pET21b plasmids containing different triggers. (N=3, t-distribution two-sided 90% confidence interval). (**b**) Impact of the "control gRNA" (i.e. the AsCas12a handle at the 3' end of the SD gRNA that is used to remove the transcriptional terminator) on the fluorescence for the control trigger and the trigger for SD gRNA t1 version C. ctrl: control (N=3, t-distribution two-sided 90% confidence interval). (**c**) Fluorescence time traces of three technical replicates of cultures as shown in (a) with and without induction of dCas12a by IPTG. (**d**) OD and fluorescence time traces for three technical replicates of two different SD gRNA plasmids (target t1) with and without induction of dCas12a by IPTG. (**e**) Fold repression for different SD gRNA versions with the control trigger at different time points. (N=4, t-distribution two-sided 90% confidence interval) (**f**) Comparison of repression by SD gRNA t1 version C for two different IPTG concentrations. trig=trigger (N=4, t-distribution two-sided 90% confidence interval) Source data are provided as a Source Data file.

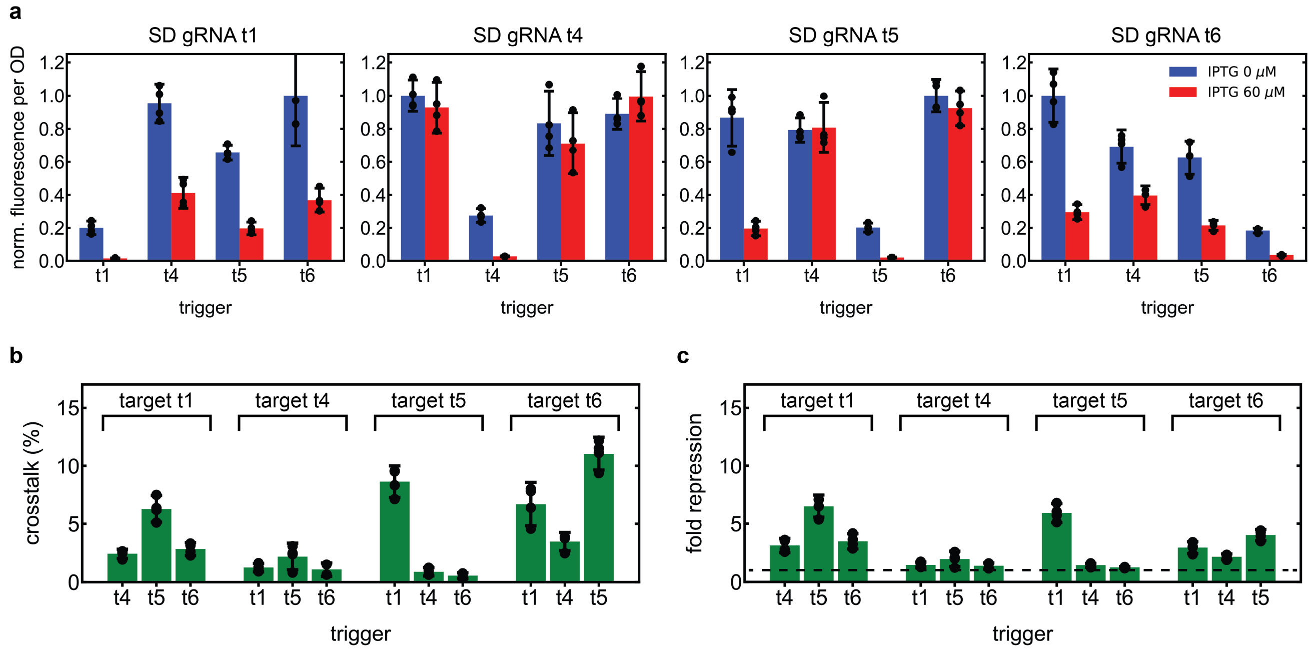


**Supplementary Figure 9. Analysis of crosstalk for in vivo SD gRNAs.** (**a**) Fluorescence per OD with and without induction of dCas12a by IPTG for the four in vivo SD gRNAs (version C) analyzed four hours after induction. (N=4, t-distribution two-sided 90% confidence interval) (**b**) Relative crosstalk between SD gRNAs and non-cognate triggers four hours after induction. The percentage crosstalk was determined by the fold repression for the cognate triggers divided by the fold repression for the shown trigger minus the fold repression for the cognate trigger divided by the fold repression for the control trigger. (N=4, t-distribution two-sided 90% confidence interval) (**c**) Fold repression for non-cognate triggers four hours after induction. The dashed line marks 1-fold repression (i.e. no repression). (N=4, t-distribution two-sided 90% confidence interval) Source data are provided as a Source Data file.

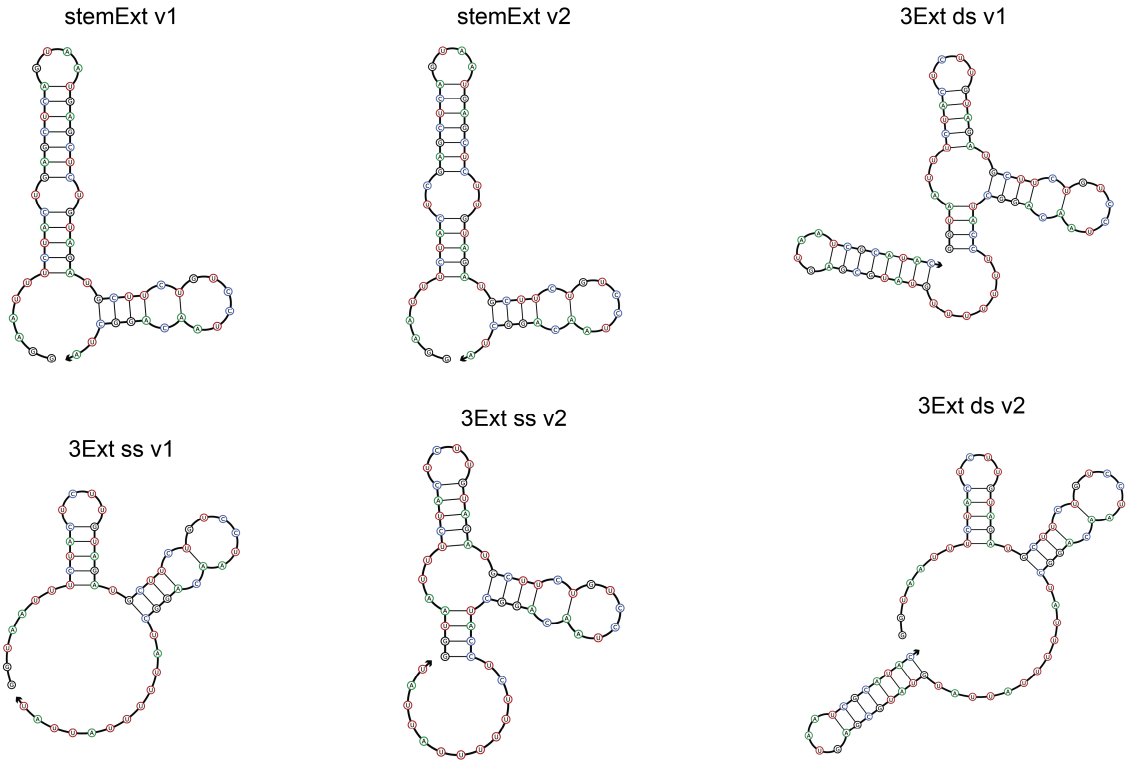


**Supplementary Figure 10. Predicted folding (minimum free energy structures) of stem extension and 3' extension Cas12a gRNAs [1].** Two tested sequences for extension of the stem and four sequences for extension at the 3' end are shown. The tested sequences do not exhaust the possibilities for extending the gRNA at these positions, so it is quite possible that some extension scheme exists that leads to functional gRNAs even when extending at these positions. For our purposes, testing a single target sequence is sufficient, since we desire a universal extension scheme that is valid for all possible target sequences.

5Ext ss v1

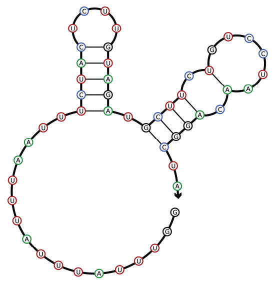

5Ext ss v2

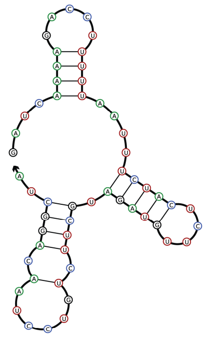

5Ext ss v3

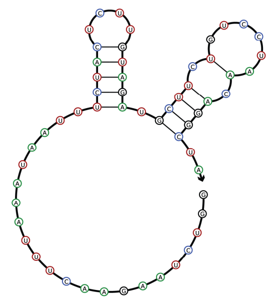

5Ext ds v1

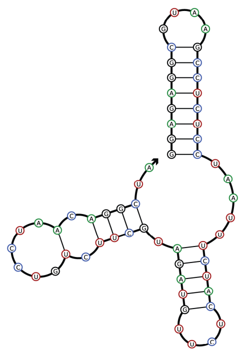

5Ext ds v2

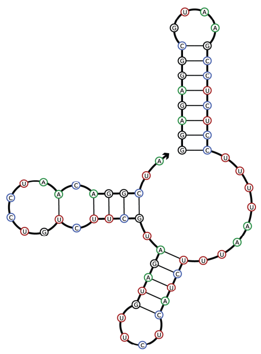

5Ext ds v3

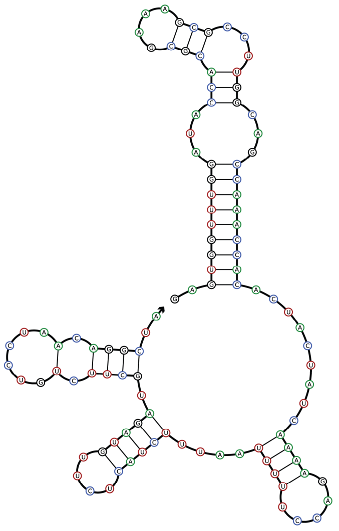

**Supplementary Figure 11. Predicted folding of 5' extension Cas12a gRNAs [1].**

Predicted secondary structures of six gRNAs with a t1 target domain that were extended at the 5' end. The cutting activity is shown in **Fig. 1c**. The cutting activity for variants with a t2 target sequence are shown in **Supplementary Figure 2**. We tested three different fully single-stranded extensions and three different partially double stranded extensions.

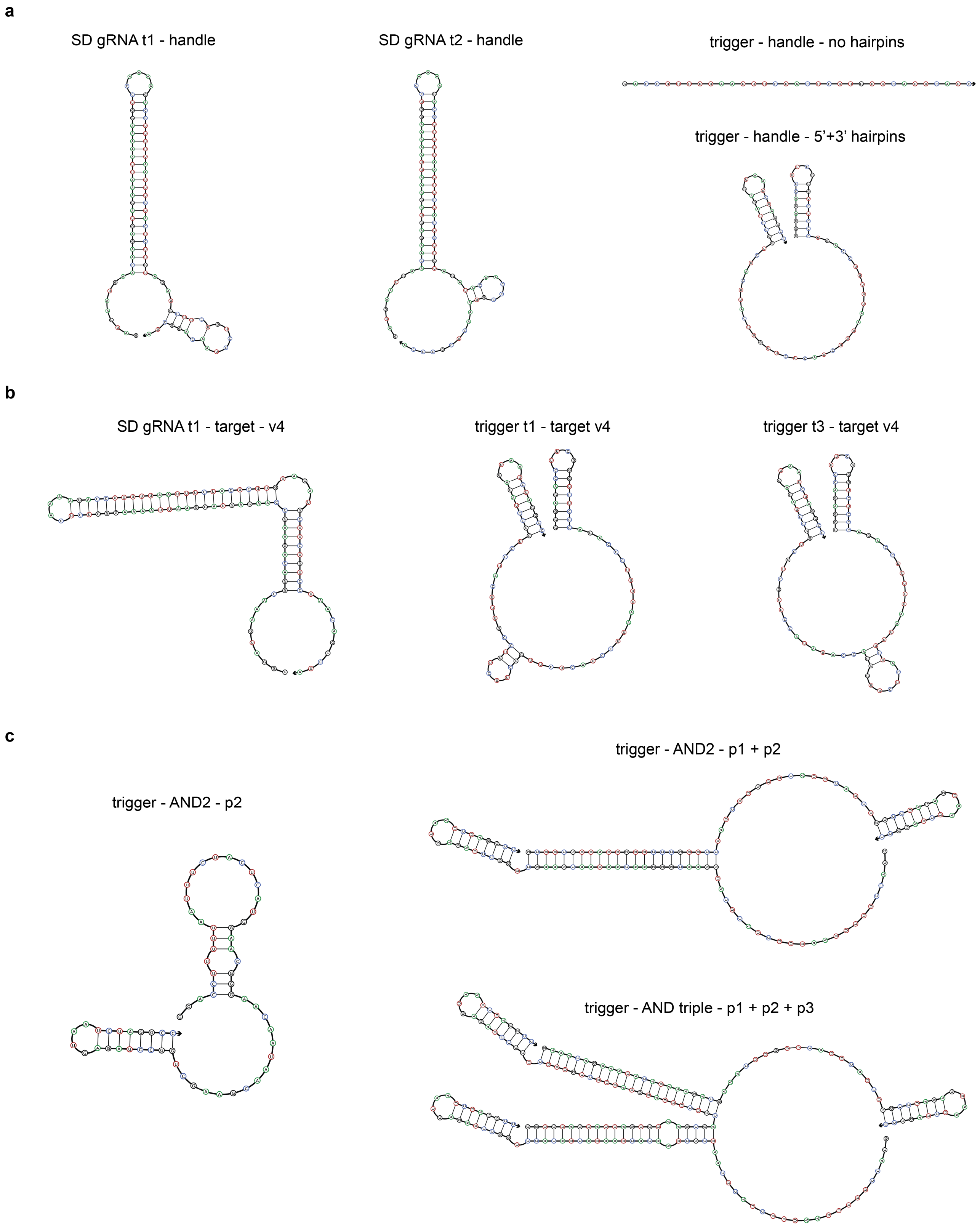


**Supplementary Figure 12. Predicted folding of selected in vitro SD gRNAs and triggers.** (**a**) Handle-based gRNA designs. (**b**) Target-based designs. (**c**) AND gates [1].

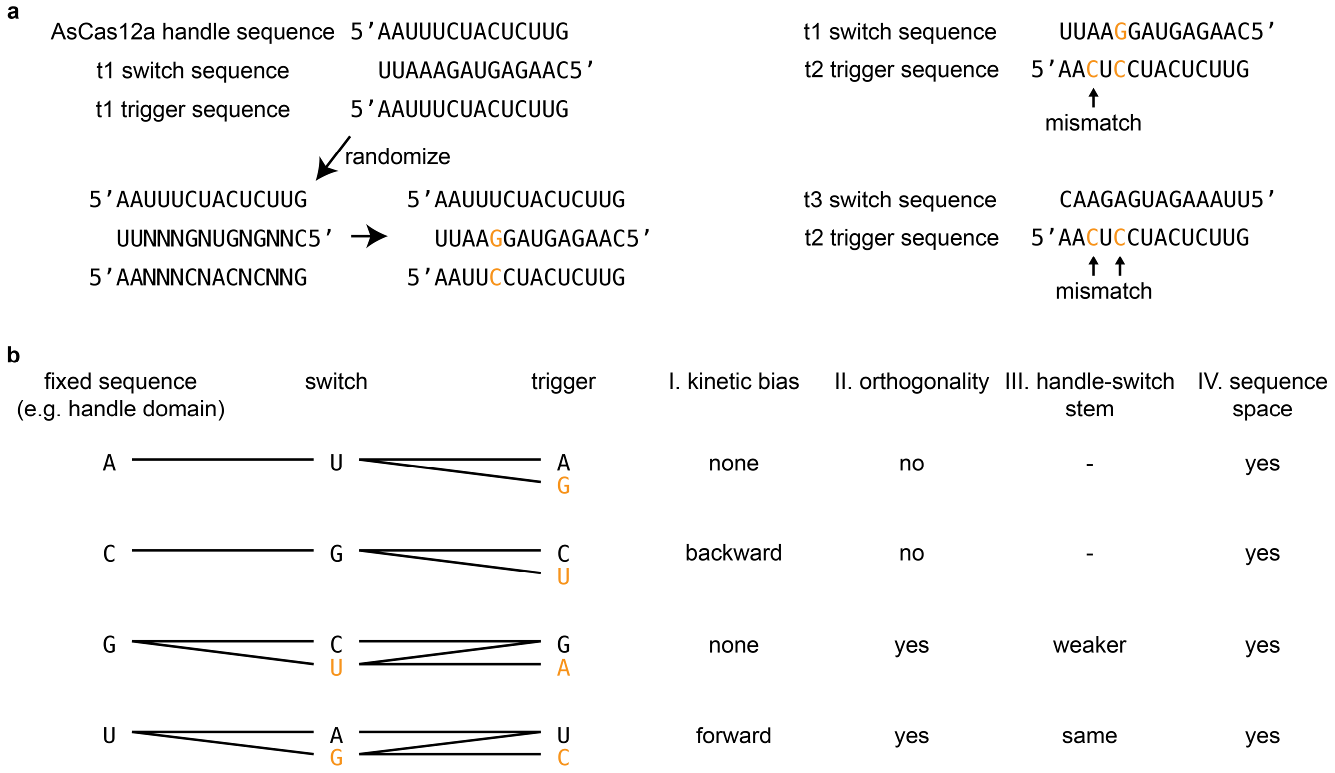


**Supplementary Figure 13. Expected impact of GU base pairs on the design of SD gRNAs.** (**a**) GU pairs in the target-based SD gRNA design. (**b**) Expected properties of GU pairs depending on the base that is replaced.

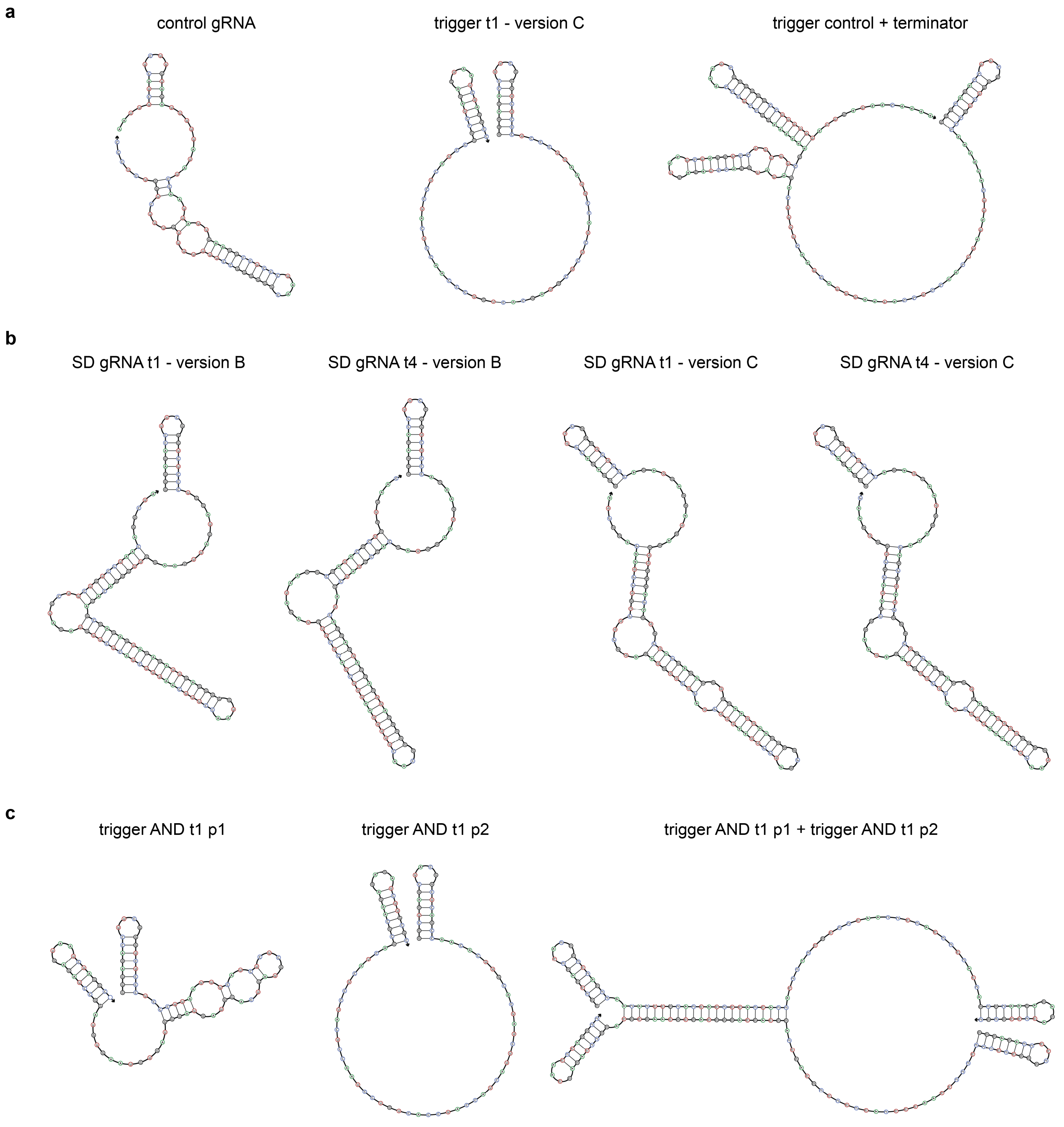


**Supplementary Figure 14. Predicted folding of in vivo SD gRNAs and triggers.** (**a**) The AsCas12a handle at the 3' end of the SD gRNA that is used to remove the transcriptional terminator, a trigger for SD gRNA t1 – version C, and the control trigger with its terminator. (**b**) SD gRNA versions B and C for two different target sequences. SD gRNA t4 – version B was designed by NUPACK, but not tested in this paper. (**c**) Individual triggers p1 and p2 of the AND gate and their hybridization product.

# Supplementary Notes

## Supplementary Note 1 – Design of stem extension and 3' extension Cas12a gRNAs

**Supplementary Figure 10** shows the predicted minimum free energy structures of the stem extension and 3' extension gRNAs.

For the stem extension, we tried extending a truncated variant of the AsCas12a gRNA handle that was previously identified as functional (v1) and the full handle by a hairpin terminated by a tetraloop (v2)[2]. Unlike for Cas9, the Cas12a handle does not extend past the protein [3, 4]. Therefore, extending the handle at the stem might cause steric hindrances that interfere with Cas12a binding, which could explain why these extensions are completely nonfunctional (**Fig. 1b**).

For the 3' extension, we extended the gRNA by two different unstructured single stranded extensions and two different partially double stranded extensions. Version "ss v1" has a 20 nt target domain followed by a 10 nt single stranded extension. Version "ss v2" has a 25 nt target domain followed by a 10 nt single stranded extension. Both are less active than a regular gRNA but show reasonable activity overall. Version ss v2 was tested to ensure that mismatches between the target domain and the target after the $20^{th}$ nucleotide in version ss v1 are not responsible for reduced cutting. Version "ds v1" has a 22 nt target domain, followed by a 7 nt single stranded domain and an 8 nt stem capped by a tetraloop. Version "ds v2" has a 20 nt target domain and a 10 nt single stranded domain domain with the same hairpin. Both show only weak residual activity. Bound AsCas12a only extends until position twenty of the gRNA target domain. For both double-stranded versions the stem is therefore well outside the protein and should not cause steric hindrance when binding. As the single-stranded versions show, the single stranded domain between the target domain and the added hairpin should not be responsible for the reduced activity.

## Supplementary Note 2 – Design of 5' extension Cas12a gRNAs

**Supplementary Figure 11** shows some of the predicted minimum free energy structures of the 5' extension gRNAs.

The three single-stranded extensions test sequence variants for Cas12a gRNA processing. Version “ss v1” contains a random AU-rich 5' extension of 11 nt with two leading Gs for efficient transcription by T7 polymerase. Versions “ss v2” and “ss v3” are derived from the natural full repeat sequence of AsCas12a and FnCas12a, respectively [2]. Under the conditions tested here, all three separators show full cutting of the target, indicating substantial freedom in the choice of the 5' separator.

The three double-stranded extensions test the necessary separator length. Version “ds v1” and “ds v2” have no separator and a three U separator, respectively. Since both show efficient cutting of the target, it appears that AsCas12a-based processing of the gRNA is quite robust to structure at the 5' end. Version “ds v3” tests a larger stem at the 5' end in combination with the single-stranded extension from version “ss v2”. The separator is longer than necessary for processing to allow for a stable separator stem loop in the strand displacement application to suppress transient formation of the handle.

## Supplementary Note 3 - Design of in vitro SD gRNAs

**Supplementary Figure 12** shows some of the predicted minimum free energy structures of the in vitro SD gRNAs and triggers. The design for all SD gRNAs is performed in NUPACK [1, 5]. For some designs, the NUPACK web application is sufficient, but for more advanced designs, certain features are required that are currently only available in the offline version of NUPACK. Design files are supplied separately as text files for all designs shown in this paper. The offline design files (.np) are processed with the command "multitubedesign design_file_name.np".

**Domains of the gRNA**

The sequences of the separator (s), handle (h) and target (t) domains are:

s – CAAAAGACCUUUUU (if fixed)

h – AAUUUCUACUCUUGUAGAU

t – N20.

**Handle-based design**

For the handle-based strand displacement gRNAs, the switch domain pairs with 23 nt of the gRNA, specifically 14 nt of the handle and 9 nt of the separator. There is a trade-off between the length of this domain, the kinetics of strand displacement, and the structure of the trigger. Making the domain longer makes the SD gRNA tighter against leak. The kinetics of strand displacement are roughly quadratic in the length of the displaced region [6]. Since kinetics are fast for SD gRNAs overall, this is not a significant limitation. However, since the handle domain is fixed, making the switch longer can introduce unavoidable secondary structure to the trigger. The impact of secondary structure on strand displacement kinetics is not explored in detail in the literature, which is unfortunate since non-optimal strands are highly likely for any application involving RNAs with fixed sequences (e.g. also for mRNA sensing). In general, secondary structure can slow down hybridization kinetics significantly [7], which is why we try to avoid it as much as possible.

The gRNA handle contains a stem of 5 nt. This limits the fraction of the handle that can be paired continuously from the 5' end without GU pairs to the first 14 or 15 nt of the handle. This is the length we chose for our design. Therefore, introduction of GU pairs in the switch domain is not necessary for this design. With the use of GU pairs, pairing the entire handle would also be possible. The toehold is chosen to be 10 nt long. As the kinetics of strand displacement are extremely sensitive to toehold length, secondary structure in the toehold must be avoided [6].

The designed RNAs show no structure in the toehold and the trigger shows no secondary structure at all. The 5' and 3' hairpins do not necessarily have to be specified during the design and can be added manually without interfering with the folding of the trigger. We could exchange the target sequence to sequence t2 without having to alter the toehold or the trigger, though in general a new toehold is required for any new target sequence.

**Target-based design**

Earlier target-based designs were performed using the NUPACK web application. The final in vitro target-based design was performed using the offline version of NUPACK. The design of target-based SD gRNAs is more intricate than the design of handle-based SD gRNAs as the target domain can introduce secondary structure to the trigger. Creation of functional, orthogonal SD gRNAs requires careful consideration of the design objective.

We pair 24 nt of the handle and separator and 10 nt of the target domain of the gRNA. The length of these domains was chosen based on the leakiness of an earlier design with 18 nt handle and separator and 8 nt of target domain pairing (Supplementary Figure 5). The toehold length is 9 nt, which should maximize reaction speed as the reaction rate is expected to saturate around 7 nt toehold length [6]. 5' and 3' hairpins are added to the trigger, with a 1 nt distance between the trigger and the hairpin at the 3' end and no distance at the 5' end. The lack of distance the 5' end is intended to suppress possible leak due to toehold-free strand displacement from the loop between the switch domain and gRNA domain (**Fig. 3a**).

**GU base pairs and orthogonality**

The switch domains of the SD gRNAs and the triggers are only partially randomized (**Supplementary Figure 13a**). We first take the reverse complement of the gRNA domains that should be double-stranded in the SD gRNA as the starting sequence for the switch. Then, all As of the switch domain and all corresponding Us in the trigger are replaced by Ns. For these randomized nucleotides, the design algorithm can choose an A or a G in the switch and a U or a C in the trigger to fulfill the specified base pairing behavior.

There are two reasons for randomizing only the As: First, we observed that the algorithm had trouble converging when fully randomizing the switch domain and the trigger, indicating that a fully random switch might create too large a sequence space when simultaneously designing switches and triggers for multiple orthogonal SD gRNAs. Second, we expect that this specific choice of position for GU pairs improves the kinetics and orthogonality of the strand displacement reaction, as illustrated in the figure shown above:

When the algorithm chooses a G rather than an A to pair a U in the gRNA, it then has the option of placing a C in the trigger (**Supplementary Figure 13b**). This means that a GU pair is replaced by a GC pair during strand displacement, which we expect to bias the strand displacement in the forward direction due to the higher energy of GC pairs as compared to GU pairs [8]. Furthermore, this can create a mismatch for a second trigger if this GU pair does not occur in its corresponding SD gRNA, allowing the algorithm to introduce orthogonality even in nominally fixed sequences. The algorithm can be biased to take this choice by the optimization constraint against cross sequence interaction. The impact of different distributions of GU pairs on orthogonality for fixed sequences is shown in the figure shown above. The trigger for SD gRNA t2 has one mismatch with the handle-complementary part of the switch domain of SD gRNA t1 and two mismatches with the handle-complementary part of the switch domain of SD gRNA t3 (**Supplementary Figure 13a**).

While this additional orthogonality would also be present when randomizing Cs in the switch domain, it would not have the additional benefit of a kinetic bias in the forward direction. Since the energies of AU and GU pairs are similar, it is likely still reasonable to randomize Cs in the switch domain if additional design space is required. Replacing GC pairs by GU pairs might destabilize the switch-gRNA duplex and therefore cause leak, which is why we did not randomize Cs for our design.

In the trigger, we did not randomize Gs in accordance with the lack of randomization of Cs in the switch domain. Randomization of Cs in the trigger was avoided since it leads to a GC pair in the switch-gRNA stem being replaced by a GU pair in the trigger-switch stem during strand displacement, which could introduce a bias in the reverse direction. If additional sequence design space is required, randomizing As in the trigger is reasonable, since AU and GU pairs have similar energy. We did not use this option for our design. In general, the use of GU pairs helps to avoid secondary structure due to fixed sequences, as can be seen in the largely structure-free triggers resulting from this design approach. We have not found any previous discussion of the role of GU pairs in RNA strand displacement in the literature. A systematic investigation of the influence of GU pairs on RNA strand displacement kinetics and orthogonality would certainly be a valuable direction for future research.

Care should also be taken when designing against off-target interactions. During sequence design, we do not penalize interaction between the full SD gRNA sequence and triggers belonging to other SD gRNAs, but instead use a truncated SD gRNA in which the single-stranded part of the target domain is excluded. Unavoidable interactions between the semi-fixed sequences of the trigger and the target domain may occur. As these sequences cannot easily be altered, the only possibility for fulfilling the design objective then is to introduce secondary structure to the trigger. Thereby, specifying orthogonality between the full SD gRNA and non-cognate triggers can lead to the design of triggers that display very slow activation kinetics. The introduction of strong, unwanted secondary structure to fulfill cross-sequence interaction restrictions was observed repeatedly in different contexts, indicating that this feature must be used with caution.

Two options that are currently not available in the NUPACK web application are weighting and domain-specific sequence motif prevention, both of which are used for this design.
The design algorithm tends to introduce secondary structure to toeholds as a simple way to avoid interaction between a trigger and a toehold based on partial complementary, which is not desirable as strand displacement kinetics is very sensitive to toehold length.
By giving extra weight to the toehold when calculating the normalized ensemble defect, this behavior can be discouraged. In the toehold, which has an arbitrary sequence, we try to avoid long stretches of similar nucleotides such as AAAA. In the switch, AAAAA is part of the regular reverse complement of the gRNA. Therefore, we only suppress long stretches of Gs in the switch to stop the algorithm from introducing many sequential GU pairs which might lead to leak but suppress all quadruple base repeats in the toehold. Furthermore, we prohibit more than three weak base pairs in the toehold in a row to ensure optimal kinetics for the 9 nt toehold.

For our purposes, choosing an identical design for all SD gRNAs was reasonable. Occasionally, it might occur that there is too much secondary structure or insufficient orthogonality when using the first ten bases of the target domain for all desired SD gRNAs. In principle, it should then be possible to choose a different subdomain of the target domain or to split the target domain.

**AND gates**

The AND gate design follows the concept described in Green *et al.* and is illustrated in **Fig. 4** [9]. The trigger for the handle-based gRNA design was split in two with 20 nt on part "p1" and 14 nt on "p2". Trigger part p1 therefore contains most of the trigger necessary to displace the switch domain, while part p2 contains the toehold and a few nucleotides of the switch-displacing domain. The AND domain consists of 22 random nucleotides at the 3' end of p1 and the 5' end of p2. The AND domains are linked to the split trigger domains by a 3 nt linker.

For the triple AND gate, the splitting of the trigger is the same, but the AND domain of p1 links to a trigger part p2 that only contains two 22 nt AND domains, which again links to trigger part p3 which contains the remaining part of the split trigger.

The figure shown above shows the folding of a single trigger part and the assembled trigger for a two-input and three-input AND gate. Part p2 contains significant secondary structure in its AND domains. In the design of orthogonal AND gates, it is more important that the AND domains are orthogonal and the resulting assembled triggers are structure-free than that the individual trigger parts have no structure. The secondary structure in the AND domain of a single trigger part is quickly displaced upon binding of the other trigger part. Here, too, care must be taken with the off-target optimization. The combination of the toehold with 4 nt complementary to the switch domain causes part p2 in the two-input AND gate and part p3 in the three-input AND gate to always interact with the SD gRNA. Therefore, off-target optimization between the SD gRNA and the trigger is only performed using a truncated version of the trigger containing only the linker and the AND domain.

**AND gate mRNA sensors**

The design of the mRNA sensor is fundamentally similar to that of the AND gate discussed in the previous paragraph. The artificial trigger is trigger p1 and the mRNA is treated as trigger p2. In this case, a target-based SD gRNA was used. We expect strand displacement on the mRNA to be much slower than for a fully structure free artificial trigger and therefore attempted to minimize the part of the trigger sequence that is assigned to the mRNA. Furthermore, we prefer sensors which do not constrain the target sequence. We therefore used a fixed target sequence, namely target t2. The entire separator- and handle-cognate domains are assigned to trigger p1. The separator sequence was chosen to have a length of 14 nt wherein the first nucleotide following the loop is a G or a C and the last two nucleotides were Us. The target-cognate domain of the switch was divided into two parts with lengths of 7 nt and 6 nt, respectively. The first part was assigned to trigger p1, while the last three nucleotides of the latter were assigned to the mRNA. Trigger p2 therefore contains the AND domain, 3 nt of the target domain and a toehold-cognate domain, meaning that only 3 nt on the mRNA are fixed up to GU pairs. On the 5' and 3' side of the chosen sensed subsequence of the mRNA, thirty nucleotides were optimized to not interact strongly with the subsequence to avoid strong interference from local secondary structure on the mRNA. We did not consider global secondary mRNA structure for this design.

## Supplementary Note 4 – Transcription of RNA triggers

When transcribing completely structure-free RNA triggers, we observed that barely any correct length RNA was produced (**Supplementary Figure 4**). To improve transcription of the correct length RNA, we added a hairpin to the 3' end of the trigger, as it has been reported that a structure-free 3' end can result in aberrant products [10]. This did increase transcription in the correct length product, but a series of bands of around twice the expected length remained. For these triggers, we performed gel extraction to produce a pure trigger RNA. Adding another hairpin at the 5' end strongly decreases the transcription of incorrect-length products. Both gel-extracted trigger RNA with a 3' hairpin and trigger RNA with a 5' and 3' hairpin are fully functional. Since applications in the presence of RNases are likely to require hairpins at the ends for stability either way, we recommend adding hairpins to both ends for all trigger RNAs [11].

## Supplementary Note 5 – Details of in vivo measurements

As seen in **Supplementary Figure 8a**, the triggers alone (i.e., in the absence of SD gRNAs) are not responsible for the strong repression we observe for triggered SD gRNAs. There is, however, a significant difference in fluorescence per OD for the plasmid without any gRNA and the SD gRNA plasmids. This difference appears to be caused by the transcription of the AsCas12a handle that is used to remove the transcription terminator (**Supplementary Figure 8b**). This reduction in fluorescence is not dependent on the addition of IPTG and therefore unlikely to be due to weak repression by the nonfunctional control gRNA. The fluorescence per OD for induced cultures is slightly higher than for uninduced cultures. Since this effect is small and its size hard to predict for any specific plasmid configuration, we use the uninduced cultures as the reference for the repression values stated.

We typically take the fluorescence four hours after induction to quantify our repression and dynamic range in the main text. At this time, the cultures are in the late exponential phase (OD 0.75-1.0, **Supplementary Figure 8d**), while in the stationary phase the measurements become irreproducible. **Supplementary Figure 8c** shows that the fluorescence of technical triplicates, i.e. the same culture pipetted into adjacent wells on a 96-well plate, can show strongly divergent behavior at later times. Specifically, we observe that some cultures continue to express mVenus, while others exhibit almost constant fluorescence in stationary phase. The divergence observed is independent of the presence of SD gRNAs, and therefore also is not the result of leaky dCas12a repression.

We observe this divergence more commonly for ‘real’ triggers than for the control trigger. Therefore, the leaky repression observed for our SD gRNAs at later time points might partly be due to this effect rather than to any actual leaky repression (**Supplementary Figure 8e**). The fluorescence at 6 h for the induced SD gRNA version A is consistently lower than for the uninduced SD gRNA, likely signifying a true leak reaction, while for version C, there is a mixture of outcomes with very little or no difference and, more rarely, a complete stop of mVenus expression. This difference in behavior also shows up earlier, with version A reproducibly showing reduced expression upon induction prior to four hours, while the fluorescence of version C is the same for the induced and uninduced cultures until that point.

We chose to not exclude any of the measured samples from our analysis to avoid bias. Thereby, we likely overestimate the leak reaction and underestimate our maximum dynamic range for time points after four hours. In general, the stationary phase, which begins after four hours, is poorly defined, especially in the poor growth conditions in a 96-well plate, and therefore is not used to draw conclusions about repression efficiencies and leak.

## Supplementary Note 6 – Design of in vivo SD gRNAs

**Supplementary Figure 14** shows the predicted folding of some of the tested in vivo SD gRNAs and triggers. Transcription of SD gRNAs and triggers in vivo requires a terminator sequence. As we demonstrated in vitro, addition of a hairpin to the 3' end of SD gRNAs strongly represses Cas12a activity. To avoid this problem, the gRNA processing capabilities of Cas12a are used. Following the SD gRNA, all plasmids have a nonfunctional gRNA whose first nucleotides of the target sequence are UUUUAUAU followed by an L3S3P21 terminator. Similar to the processing of a natural CRISPR array transcript, this nonfunctional gRNA is removed from the SD gRNA by dCas12a. As a control trigger, we use an AU-rich sequence which is predicted to not interact with any of the other involved RNAs. All triggers contain a 5' hairpin, a 3' hairpin and a terminator. In the other NUPACK minimum free energy predictions shown here, the terminator is omitted. For clarity, the 5' and 3' hairpins are omitted in all schematic drawings.

**SD gRNAs version B**

For this design, the separator was constrained to have a GNRAN6 sequence. The toehold length is 12 nt, and the nucleotides 5 to 14 of the target domain are paired by the switch. The switch contains two additional nucleotides between the target-complementary and the handle-complementary domain which are meant to act as a kinetic insulator for the trigger during strand displacement. The SD gRNAs were optimized to not interact with non-cognate triggers for a concentration of 10 nM for the SD gRNA and 30 nM for the triggers. SD gRNA t4 – version B conforms to the designed sequence, but SD gRNA t1 – version B does not have a fully free kinetic insulator. Because the corresponding nucleotides are only weakly bound, we nevertheless expect them to act as kinetic insulators.

**SD gRNAs version C**

The separator has the sequence GNRAN5. The toehold length is 12 nt, and the nucleotides 5 to 14 of the target domain are paired by the switch. The switch contains two kinetic insulators between the target-complementary and the handle-complementary and within the handle-complementary domains. Due to the interruption of the handle-complementary domain by the kinetic insulator, the handle-complementary domain of the switch was extended by 3 nt. The internal loop of the kinetic insulator in the handle domain is flanked by GC pairs to suppress transient handle formation. As for version B, the SD gRNAs were optimized to not interact with non-cognate triggers for a concentration of 10 nM for the SD gRNA and 30 nM for the triggers. For this design, both SD gRNA t1 – version C and SD gRNA t4 – version C have the desired structure.

**In vivo AND gate**

The in vivo AND gate is based on version C of the SD gRNAs. The entire separator- and handle-cognate domains of the trigger are placed on part p1, the target- and toehold-cognate domains are placed on part p2. The length of the AND domain is 22 nt, as for the in vitro AND gate. The AND domain includes GU pairs to facilitate the synthesis of the DNA used for cloning. By disallowing interactions between the SD gRNA and trigger part p1, the algorithm is encouraged to introduce structure to trigger part p1. This additional structure is intended to suppress cotranscriptional binding of the trigger. The structure is displaced upon binding of trigger

part p2. The AND domain on trigger part p2 is designed to not interact with the rest of the SD gRNA, both to facilitate binding of trigger part p1 and to avoid leak by due to part p2.

# Supplementary Tables

## Supplementary Table 1 – Sequencing primers for in vivo work

| primer | sequence |
|---|---|
| plasmid: pSB3C5 | |
| VF2 | TGCCACCTGACGTCTAAGAA |
| pSB3C5 - seq 1 | GTCGGTGAACGCTCTCTA |
| pSB3C5 - seq 2 | CGTAACGTTTTGCTCACC |
| pSB3C5 - seq 3 | ATCTCGACGCTCTCCCTTATGC |
| pSB3C5 - seq 4 | GAGGTGCTGAACCTTGCC |
| pSB3C5 - seq 5 | GAAGCCCTGTGCAAGTGG |
| pSB3C5 - seq 6 | GTTATTGCTCAGCGGTGG |
| pSB3C5 - seq 7 | GGTCAGTTACTGCTGAATCATC |
| pSB3C5 - seq 8 | GCTGCAATGATACCGCGAG |
| plasmid: pET21b | |
| pET21b - seq 1 | AGTCATAAGTGCGGCGAC |
| pET21b - seq 2 | AAGGGAATAAGGGCGACAC |

# Supplementary References


1. Zadeh JN*, et al.* NUPACK: Analysis and design of nucleic acid systems. *J Comput Chem* **32**, 170-173 (2010).

2. Zetsche B*, et al.* Cpf1 Is a Single RNA-Guided Endonuclease of a Class 2 CRISPR-Cas System. *CELL* **163**, 759-771 (2015).

3. Nishimasu H*, et al.* Crystal Structure of Cas9 in Complex with Guide RNA and Target DNA. *CELL* **156**, 935-949 (2014).

4. Yamano T*, et al.* Crystal Structure of Cpf1 in Complex with Guide RNA and Target DNA. *CELL* **165**, 949-962 (2016).

5. Wolfe BR, Porubsky NJ, Zadeh JN, Dirks RM, Pierce NA. Constrained Multistate Sequence Design for Nucleic Acid Reaction Pathway Engineering. *J Am Chem Soc* **139**, 3134-3144 (2017).

6. Zhang DY, Winfree E. Control of DNA Strand Displacement Kinetics Using Toehold Exchange. *J Am Chem Soc* **131**, 17303-17314 (2009).

7. Gao Y, Wolf LK, Georgiadis RM. Secondary structure effects on DNA hybridization kinetics: a solution versus surface comparison. *Nucl Acids Res* **34**, 3370-3377 (2006).

8. Serra MJ, Turner DH. Predicting thermodynamic properties of RNA. *Methods Enzymol* **259**, 242-261 (1995).

9. Green AA, Kim J, Ma D, Silver PA, Collins JJ, Yin P. Complex cellular logic computation using ribocomputing devices. *Nature* **548**, 117-121 (2017).

10. Triana-Alonso FJ, Dabrowski M, Wadzack J, Nierhaus KH. Self-coded 3'-extension of run-off transcripts produces aberrant products during in vitro transcription with T7 RNA polymerase. *J Biol Chem* **270**, 6298-6307 (1995).

11. Smolke CD, Keasling JD. Effect of gene location, mRNA secondary structures, and RNase sites on expression of two genes in an engineered operon. *Biotechnol Bioengin* **80**, 762-776 (2002).
