# Supplementary material for: Switching the activity of Cas12a using guide RNA strand displacement circuits
Source: Nat Commun. 2019 May 7;10:2092. doi: 10.1038/s41467-019-09953-w (PMC6504869; doi:10.1038/s41467-019-09953-w)
Supplement: Supplementary file 2 — Description of Additional Supplementary Files [file 41467_2019_9953_MOESM2_ESM.pdf]

Description of Additional Supplementary Files

File Name: Supplementary Data 1
Description: DNA targets used for cutting assays, DNAs used for the production of transcription templates, and transcription templates used to produce RNA for in vitro experiments

File Name: Supplementary Data 2
Description: RNA sequences for in vitro experiments

File Name: Supplementary Data 3
Description: RNA sequences for in vivo experiments

File Name: Supplementary Data 4
Description: Plasmids used for in vivo experiments

File Name: Supplementary Software 1
Description: NUPACK files are supplied as .np files (for the offline version) or as .txt files (for the online version) in the Supplementary Software file. It contains the following designs:

- SD gRNA - handle.txt: handle-based SD gRNA design (Figure 2)
- SD gRNA - target - v1.txt: v1 of target-based SD gRNA designs (Supporting Figure 5)
- SD gRNA - target - v2.txt: v2 of target-based SD gRNA designs (Supporting Figure 5)
- SD gRNA - target - v3 - t1.txt:SD gRNA t1 v3 of target-based SD gRNA designs (Supporting Figure 5)
- SD gRNA - target - v3 - t2.txt:SD gRNA t2 v3 of target-based SD gRNA designs (Supporting Figure 5)
- SD gRNA - target - v3 - t3.txt:SD gRNA t3 v3 of target-based SD gRNA designs (Supporting Figure 5)
- SD gRNA - target - v3 - all.txt:v3 of target-based SD gRNA designs (Supporting Figure 5)
- SD gRNA - target - v4.np: v4 of target-based SD gRNA designs (Figure 3, Supporting Figure 5)
- SD gRNA AND1.txt: single in vitro two-input AND gate (Figure 4)
- SD gRNA AND2.txt: orthogonal in vitro two-input AND gate (Figure 4)
- SD gRNA AND - triple.txt: three-input in vitro AND gate (Figure 4)
- two-input AND gate mCerulean sensor.np: design file for AND gate for mCerulean sensing (Supporting Figure 7)
- SD gRNA - in vivo - version B.np: design file for in vivo SD gRNA version B (Figure 5)
- SD gRNA - in vivo - version C.np: design file for in vivo SD gRNA version C (Figure 5)
- SD gRNA - in vivo - AND gate.np: design file for in vivo AND gate (Figure 6)
