# Supplementary material for: Switching the activity of Cas12a using guide RNA strand displacement circuits
Source: Nat Commun. 2019 May 7;10:2092. doi: 10.1038/s41467-019-09953-w (PMC6504869; doi:10.1038/s41467-019-09953-w)
Supplement: Supplementary file 8 — Reporting Summary [file 41467_2019_9953_MOESM8_ESM.pdf]

nature research

Corresponding author(s): Friedrich C. Simmel

Last updated by author(s): Mar 26, 2019

Reporting Summary

Nature Research wishes to improve the reproducibility of the work that we publish. This form provides structure for consistency and transparency in reporting. For further information on Nature Research policies, see Authors & Referees and the Editorial Policy Checklist.

Statistics

For all statistical analyses, confirm that the following items are present in the figure legend, table legend, main text, or Methods section.

n/a	Confirmed	
☐	☒	The exact sample size (n) for each experimental group/condition, given as a discrete number and unit of measurement
☐	☒	A statement on whether measurements were taken from distinct samples or whether the same sample was measured repeatedly
☒	☐	The statistical test(s) used AND whether they are one- or two-sided<br>*Only common tests should be described solely by name; describe more complex techniques in the Methods section.*
☒	☐	A description of all covariates tested
☒	☐	A description of any assumptions or corrections, such as tests of normality and adjustment for multiple comparisons
☐	☒	A full description of the statistical parameters including central tendency (e.g. means) or other basic estimates (e.g. regression coefficient) AND variation (e.g. standard deviation) or associated estimates of uncertainty (e.g. confidence intervals)
☒	☐	For null hypothesis testing, the test statistic (e.g. F, t, r) with confidence intervals, effect sizes, degrees of freedom and P value noted<br>*Give P values as exact values whenever suitable.*
☒	☐	For Bayesian analysis, information on the choice of priors and Markov chain Monte Carlo settings
☒	☐	For hierarchical and complex designs, identification of the appropriate level for tests and full reporting of outcomes
☒	☐	Estimates of effect sizes (e.g. Cohen's d, Pearson's r), indicating how they were calculated

Our web collection on statistics for biologists contains articles on many of the points above.

Software and code

Policy information about availability of computer code

Data collection	Microplate reader data were recorded using the commercial Omega software (version 5.11 R4). Gel images were recorded using the commercial Typoon software (version 1.0). In vitro fluorescence data were recorded using the commercial iQ5 software (version 2.1).
Data analysis	Python 3.7 was used to plot data. Fiji (ImageJ 1.52i) was used to analyze gels. The design of sequences was performed using NUPACK version 3.2.2 and the NUPACK web application (available at http://www.nupack.org).

For manuscripts utilizing custom algorithms or software that are central to the research but not yet described in published literature, software must be made available to editors/reviewers. We strongly encourage code deposition in a community repository (e.g. GitHub). See the Nature Research guidelines for submitting code & software for further information.

Data

Policy information about availability of data

All manuscripts must include a data availability statement. This statement should provide the following information, where applicable:

- Accession codes, unique identifiers, or web links for publicly available datasets
- A list of figures that have associated raw data
- A description of any restrictions on data availability

All the conclusions drawn in this paper are derived from data shown either in the main text or in the Supplementary Information. The sequences for all used components can be found in Supplementary Data 1 to 4 and Supplementary Table 1. The source data underlying Figures 2 to 6 and Supplementary Figures 3 and 5 to 9 are provided as a Source Data file. It is also available from the corresponding author upon reasonable request.

Field-specific reporting

Please select the one below that is the best fit for your research. If you are not sure, read the appropriate sections before making your selection.

☒ Life sciences ☐ Behavioural & social sciences ☐ Ecological, evolutionary & environmental sciences

For a reference copy of the document with all sections, see nature.com/documents/nr-reporting-summary-flat.pdf

Life sciences study design

All studies must disclose on these points even when the disclosure is negative.

Sample size	Sample size was not determined statistically. The sample sizes (N=3 or N=4) are based on typically accepted literature values and are stated in the Figure legends.
Data exclusions	We did not pre-establish exclusion criteria. For the measurements in E. coli, no data were excluded. For in vitro data, we discarded measurements as a whole if there were obvious problems (e.g. no cutting for the positive control in a gel assaying cutting).
Replication	We repeated all experiments at least three times. All experiments could be replicated.
Randomization	This study only contains in vitro data and measurements of E. coli cultures. Therefore, sample randomization was not relevant to this study.
Blinding	This study only contains in vitro data and measurements of E. coli cultures. Therefore, blinding was not relevant to this study.

Reporting for specific materials, systems and methods

We require information from authors about some types of materials, experimental systems and methods used in many studies. Here, indicate whether each material, system or method listed is relevant to your study. If you are not sure if a list item applies to your research, read the appropriate section before selecting a response.

Materials & experimental systems

n/a	Involved in the study	
☒	☐	Antibodies
☒	☐	Eukaryotic cell lines
☒	☐	Palaeontology
☒	☐	Animals and other organisms
☒	☐	Human research participants
☒	☐	Clinical data

Methods

n/a	Involved in the study	
☒	☐	ChIP-seq
☒	☐	Flow cytometry
☒	☐	MRI-based neuroimaging
